# Analysis of Wall Thickness and Absorption Characteristics of Ammonium Nitrate(V) from Various Sources

**DOI:** 10.3390/ma17184618

**Published:** 2024-09-20

**Authors:** Andrzej Biessikirski, Grzegorz Piotr Kaczmarczyk, Łukasz Kuterasiński, Grzegorz Machowski, Agnieszka Stopkowicz, Małgorzata Ruggiero-Mikołajczyk

**Affiliations:** 1 Faculty of Civil Engineering and Resource Management, AGH University of Krakow, 30-059 Krakow, Polandastopkow@agh.edu.pl (A.S.); 2 Jerzy Haber Institute of Catalysis and Surface Chemistry, Polish Academy of Sciences, 30-239 Krakow, Poland; lukasz.kuterasinski@ikifp.edu.pl (Ł.K.); malgorzata.ruggiero-mikolajczyk@ikifp.edu.pl (M.R.-M.); 3 Faculty of Geology, Geophysics, and Environmental Protection, AGH University of Krakow, 30-059 Krakow, Poland; machog@agh.edu.pl

**Keywords:** ammonium nitrate(V), prill, fertilizer, tomography, wall thickness, S_BET_

## Abstract

This study investigates the wall thickness and specific surface area (S_BET_) of ammonium nitrate(V) samples of varying provenance. The research focuses on both fertilizer-grade ammonium nitrate(V) and three porous prill samples obtained from different manufacturers. The samples were analyzed using tomography scanning and two distinct porosimetry methods. The wall thickness analysis revealed that fertilizer-grade ammonium nitrate(V) possesses thicker walls, ranging from 0.05 to 0.40 mm, compared to porous prill-type ammonium nitrate(V), which predominantly exhibited wall thicknesses between 0.05 and 0.025 mm, with occasional thicker regions up to 0.040 mm. These variations in wall thickness are likely attributable to differences in manufacturing processes and prilling conditions specific to the ammonium nitrate(V) porous prill-type samples. The specific surface area (S_BET_), derived from nitrogen adsorption measurements, indicated that the samples exhibited surface areas ranging from 0.011 to 0.466 m^2^·g, suggesting that these samples do not exhibit particularly high absorption capacities. However, the S_BET_ values obtained from the mercury intrusion method suggested significantly higher absorption capacities, falling within the range of 4.87–18.29 m^2^·g. These findings suggest that mercury porosimetry may provide a more accurate assessment of the porosity and absorption potential of ammonium nitrate(V) samples.

## 1. Introduction

Ammonium nitrate(V) (AN) is a colorless or white-to-gray crystalline substance or an odorless bead, possessing a density of 1.725 g·cm^−3^. It has a melting point of 169.5 °C and a boiling point of 210 °C, at which it decomposes with the release of nitrous oxide [1,2]. Upon chlorination, AN forms chloramines and is incompatible with several substances, including acetic acid, acetic anhydride, hexamethylene tetramine acetate, mixtures of nitric acid and ammonia, aluminum, calcium nitrate, formamide, various metals, alkali metals, and combustible materials [1]. Due to its chemical properties, AN has diverse applications. It is widely used in fertilizers, pyrotechnics, herbicides, and insecticides and plays a significant role in the production of nitrous oxide [3]. AN also serves as an absorbent for nitrogen oxides, a component in freezing mixtures, an oxidizing agent in rocket propellants, and a nutrient source for yeast and antibiotic production [3]. Moreover, it is a major component in energetic materials [4], as well as it is present in the food industry as a compound that influences flavor and the characteristic food color [3]. Depending on its intended use, AN is manufactured in various forms, including granules, prills, mini-prills, and aqueous solutions [5,6,7]. According to Leonard et al., the global annual production of AN was evaluated at 15.3 million tons; however, Oxley states that the general worldwide capacity in 2020 was evaluated at 63 million tons [8,9].

The application of ammonium nitrate (AN) is influenced by its absorption capabilities and porosity which are, critical factors in its performance across various uses. Porosimetry, the study of pore characteristics, is commonly evaluated using methods such as mercury intrusion porosimetry (MIP) and nitrogen adsorption techniques. MIP is a widely utilized method due to its ability to characterize a broad range of pore sizes [10]. However, this technique involves the injection of pressurized mercury into the sample, which can potentially disrupt the microstructure, leading to inaccurate results. Therefore, careful handling and measurement are essential to minimize such risks. Additionally, the presence of ink-bottle pores, which are narrow entrances leading to wider internal cavities, can also cause inaccuracies in MIP results [10,11,12].

On the other hand, the nitrogen adsorption method is a standard approach for measuring the specific surface area and pore size distribution of materials. This method operates on the principle that nitrogen gas condenses in micropores when the relative pressure (P/P0) reaches or exceeds 0.4, owing to capillary condensation [13]. However, some researchers have noted that while both MIP and nitrogen adsorption methods effectively evaluate pore distribution and the development of fractures, minerals, and other microscopic deformations, they may lack quantitative characterization and a comprehensive representation of the pore-fracture structure [14,15,16,17].

The majority of the literature on AN focuses on scanning electron microscopy (SEM) analyses [5,18,19], with limited studies dedicated to its porosity [4,9,20,21]. For instance, Miyake et al. examined various pore characteristics, including mode, median, and average pore diameters, total pore volume, and total pore area of six different AN samples using mercury porosimetry. They assumed a cylindrical shape for the pores within the particles and explored the relationship between porosity and the blasting properties of energetic materials [20,21]. Zawadzka-Małota and Maranda investigated the porosity of AN by measuring its actual density using the helium density method, reporting porosity values ranging from 44.1% to 60.2% for different sources of AN [4]. Additionally, Leonard et al. performed a structural and morphological quantitative 3D characterization of ammonium nitrate prills using X-ray computed tomography [9].

As evident from the literature, there is a scarcity of detailed information on the porosity analysis of AN from various sources. The primary objective of this study is to demonstrate the research on SEM and tomographic characterization of ammonium nitrate(V). In addition, by investigating the porosity of ammonium nitrate (AN) samples using two different and widely utilized evaluation methods, we aim to determine the most suitable approach for the characterization of AN samples. Furthermore, through the analysis of wall thickness, we seek to elucidate how the manufacturing process influences the crystal structure of ammonium nitrate(V), which plays a crucial role in its performance in various applications, such as mining and agriculture.

## 2. Materials and Methods

### 2.1. Materials

In this research, one sample of granule ammonium nitrate(V) and three samples of prilled ammonium nitrate(V) were analyzed.

Sample 1: This fertilizer-grade ammonium nitrate (AN-F) was supplied by Anvil S.A., Włocławek, Poland. AN-F comprised 34.0% nitrogen, equally distributed between nitrate and ammoniacal nitrogen forms, and included 0.2% magnesium. Magnesium was present in the form of magnesium nitrate. The prill size varied from 1 mm to 3 mm. The density of the AN-F ranged from 920 to 1000 kg·m^−3^. The water content in the granule was approximately 0.4%.

Sample 2: Ammonium nitrate(V) porous prilled size 8 (AN-PP8-1) was produced by Yara International ASA, Szczecin, Poland. It had a purity of 99.5%. The remaining 0.5% was coating agents. This sample contained approximately 35.0% nitrogen. The average prill diameter was 1 mm. The bulk density of AN-PP8-1 was 820 kg∙m^−3^. The water content did not exceed 0.3%.

Sample 3: Ammonium nitrate(V) porous prilled size 7 (AN-PP7) was also manufactured by Yara International ASA, Szczecin, Poland. This prill demonstrated a purity of 99.5% and a nitrogen content of approximately 35.0%. The average prill diameter was 0.8 mm. The bulk density was approximately 740 kg∙m^−3^. The water content remained below 0.3%.

Sample 4: Ammonium nitrate(V) porous prilled size 8 (AN-PP8-2), supplied by a different manufacturer, exhibited 98.0% purity. The remaining 2.0% were coating agents and organic additives. The organic additives were approximately (0.15%). Sample 4 contained approximately 35.0% nitrogen. AN-PP-8-2 was in the prill form. The prill diameter averaged 1 mm. The bulk density was approximately 800 kg∙m^−3^. The water content did not exceed 0.5.

### 2.2. Methods

A tomographic scan was conducted using a GE Phoenix v|tome|x M device (General Electric Company, Hürth, Germany), utilizing X-ray spectroscopy technology.

AN samples were mounted on a low-absorption foam substrate using adhesive contact. The foam, along with the sample, was subsequently positioned within the tomographic chamber. The scans were performed with a voxel size of 4.23 µm^3^, under a radiation power of 40 kV and 120 mA, with appropriate detector settings. Each 360° scan required approximately 60 min, resulting in the acquisition of 2600 projections.

**Figure 1:**
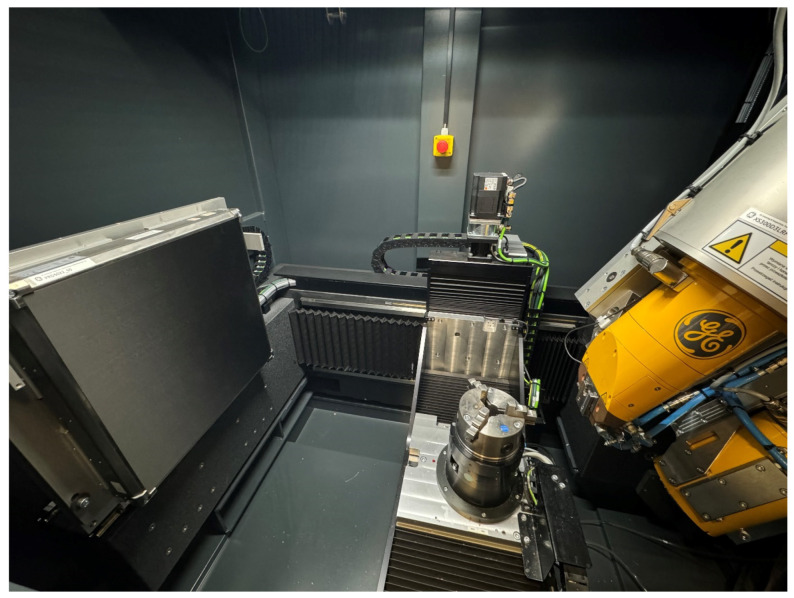
The illustration of a tomographic chamber.

The researched sample in a tomographic chamber is presented in Figure 1.

The next phase involved reconstructing the three-dimensional volume from the obtained 2D projections and using Phoenix datos|x 2 rec software (version 2.7.2 RTM 2019). A detailed description of the procedure was presented in [22].

The initial step in processing the reconstructed data involved determining the value at which the material boundary occurs. This value was individually determined for each sample based on the histogram. A wall thickness analysis was performed on the reconstructed data using the Ray method. The Ray method involves computing the thickness by projecting a ray normal to the surface of the model toward the material side. The point where this ray intersects the opposite surface is considered the hit point. The distance between the starting point and the hit point is referred to as the “ray thickness”.

Porosimetry tests were conducted in Autosorb-1 by the Former Quantachrome Instruments (Boynton Beach, FL, USA) device and AutoPore IV 9520 produced by Micrometrics (Atlanta, GA, USA).

The porosimetry evaluation using the Autosorb-1 was performed through low-temperature nitrogen sorption at −196 °C. The specific surface area (S_BET_) was determined using the Brunauer–Emmett–Teller (BET) model. The pore size distribution was evaluated by Barrett, Joyner, and Halenda’s (BJH) method; however, due to obtained S_BET_ results, the authors decided not to publish them. Before measurement, each sample was degassed at room temperature for 18 h. The temperature was selected based on the potential influence of the sample, as decomposition gases could contaminate the equipment’s sensors.

The Mercury Intrusion Porosimetry (MIP) method is extensively employed in geological analysis of rocks due to its ability to provide comprehensive information on a sample’s pore size distribution, total pore volume, porosity, skeletal, and apparent density, as well as specific surface area [23,24,25]. The technique is based on the principle that mercury acts as a non-wetting liquid when in contact with most solids. Consequently, it does not infiltrate the pores or fractures of these materials unless external pressure is applied. The diameter of the pores intruded by mercury is inversely related to the applied pressure. The pressure (p) is determined by the contact angle (θ_Hg_) between mercury and the porous material, the gas/liquid surface tension of mercury (ɣ_Hg_), and the pore diameter (D). This relationship is governed by the Young–Laplace law, specifically described by the Washburn equation for cylindrical pores, Equation (1) [26,27].

(1) $$D=-\frac{4{ɣ}_{\mathrm{Hg}}\cdot {\cos\theta }_{\mathrm{Hg}}}{P}$$

Before the measurements, all samples were dried for 24 h at 110 °C to remove any existing water content. After drying samples were degassed at 25–27 °C.

The MIP equipment parts were constructed from corrosion-resistant glass and steel, and the measurement material did not adversely affect the instruments. No indications of improper equipment or sensor operation were observed.

A key point in porosimetry evaluation is the measurement uncertainty of both methods. De Lang et al. conducted a detailed analysis of the uncertainties associated with the adsorptive characterization of porous solids, focusing primarily on the determination of pore volume using the Brunauer–Emmett–Teller (BET) method. They identified that major uncertainties in adsorption measurements could be mitigated by increasing the sample mass, enhancing measurement accuracy, or optimizing the ratio between manifold and cell volume to a range of 2–3. Their study demonstrated that using larger cell volumes or smaller sample masses can significantly increase or artificially introduce hysteresis between adsorption and desorption isotherms [28]. In the context of microporous materials, De Lang et al. concluded that when the relative pressure (defined as the ratio of the pressure to the saturated vapor pressure of nitrogen) is below 0.9, the relative uncertainty in pore volume determination decreases [28]. Furthermore, when evaluating the BET surface area, they emphasized that the primary source of uncertainty, as supported by various studies [29,30,31,32], is linked to the choice of the fitting model used in the analysis. De Lang et al. also noted that increasing the degrees of freedom by using at least five data points and excluding data points near saturation significantly reduces errors in the analysis. However, the literature lacks explicit guidelines on which data points should be excluded a priori when working in the low relative pressure range, where surface heterogeneity can strongly interfere with measurements. To address this, De Lange et al. suggested using standardized residuals to identify boundaries for exclusion. Additionally, they reported that the 95% confidence limits for Barrett–Joyner–Halenda (BJH) pore size distributions are severely affected under certain conditions [28].

Contrary to nitrogen absorption, the MIP is a relatively simple method for evaluating pore size distribution in solids. However, the accuracy of the results may not be as reliable as they appear. The pore size evaluation in MIP is based on the Washburn equation, which assumes that the pore system consists of uniformly cylindrical pores that are fully accessible from the crystal surface of the sample. This implies that no distinction is made between mercury intrusion into a single long pore, a single elongated cylindrical pore, or a series of identical short cylindrical pores. As Diamond concluded, this assumption holds for only a limited number of materials [12].

It is important to note that the MIP method exhibits greater measurement uncertainty when applied to hydrated samples or those with elevated water content, due to the limitations of the Washburn equation. In such cases, the measured pore size may be one to two orders of magnitude smaller than the actual pore size. However, in this study, the drying procedure and low laboratory humidity (~40%) minimized the influence of water content, making its impact on the measurements negligible.

The oil absorption capacity was assessed by incrementally adding 0.25 cm^3^ of fuel oil to a 100 g sample of ammonium nitrate(V). When the free, unbound fuel oil became visible within the mixture the research was stopped.

## 3. Results and Discussion

**Figure 2:**
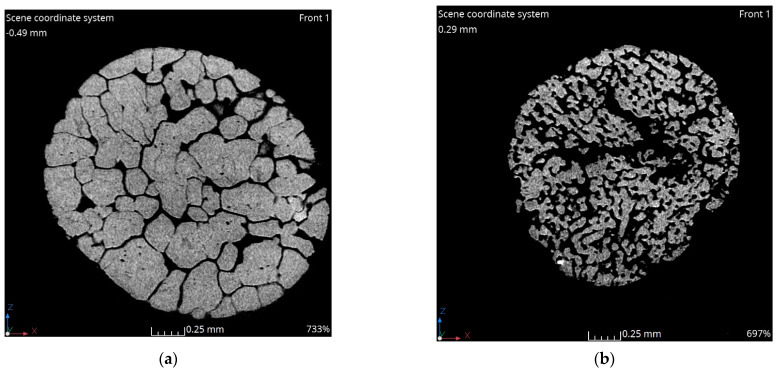

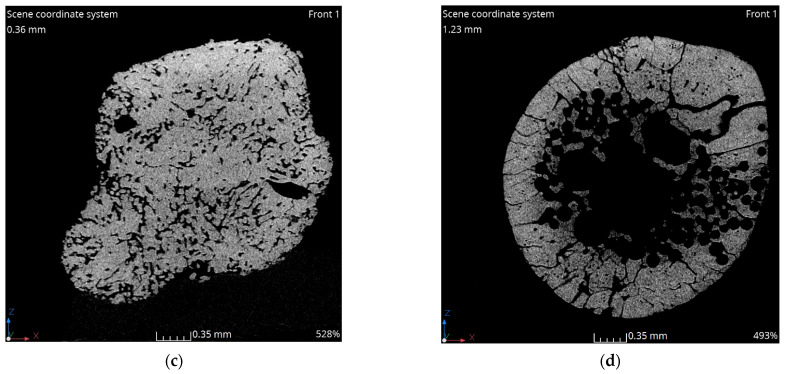
Tomography XY cross-sectioned pictures of various ammonium nitrate(V) prills and granules: (**a**) AN Sample 1, (**b**) AN Sample 2, (**c**) AN Sample 3, and (**d**) AN Sample 4 [21].

**Figure 3:**
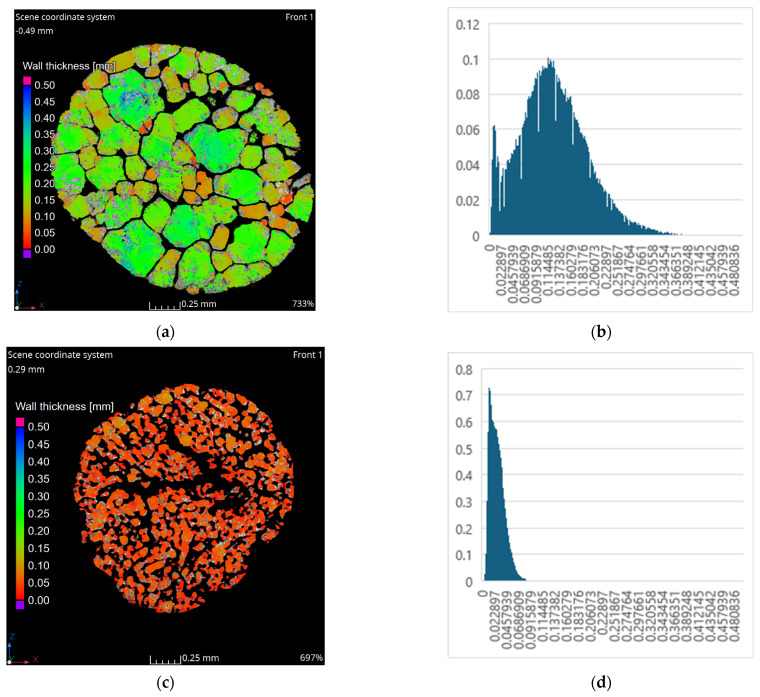

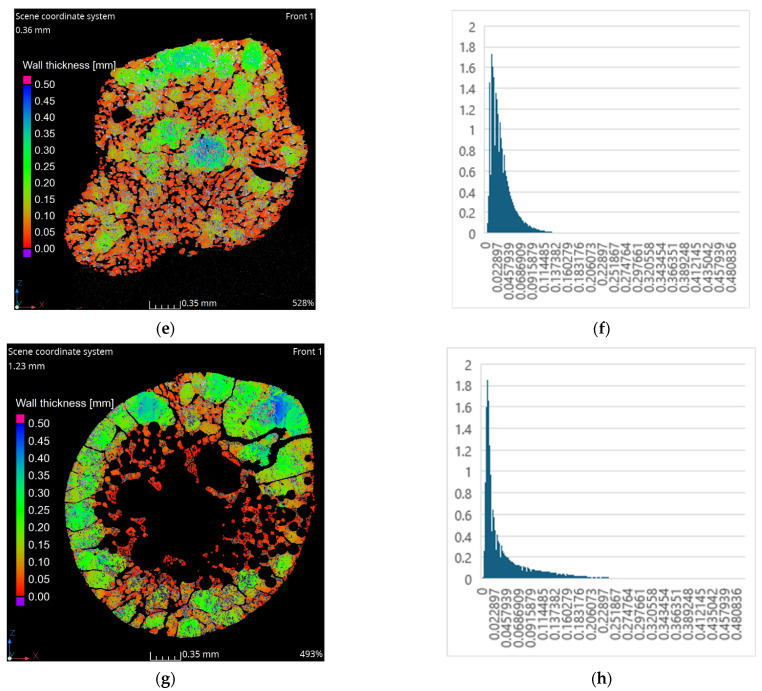
Tomography XY cross-sectioned data of various ammonium nitrate (V) prills and granules and pore size distribution: (**a**) AN Sample 1, (**b**) pore size distribution sample 1, (**c**) AN Sample 2, (**d**) pore size distribution sample 2, (**e**) AN Sample 3, (**f**) pore size distribution sample 3, (**g**) AN Sample 4, and (**h**) pore size distribution sample 4.

In previous research, it was indicated that researched ammonium nitrate(V) scans, Figure 2, showed similar morphology in the same part; however, the distinct differences between samples were visible. In general, it was established that almost all tested samples except fertilizer-grade ammonium nitrate(V)—sample 1, were characterized by the open-porosity. Fertilizer-grade ammonium nitrate(V) had several channels that were meandered throughout the entire granule. Additionally, it was indicated that the closed porosity of sample 1 was due to the presence of the coating agent [22]. Porous prill samples samples 2–4 were characterized by the open-porosity and rough internal structures. Pores were uniformly distributed and were characterized by local elongation in one direction (sample 3) and the presence of the central cavity (sample 4). The central cavity was probably a result of the production process and longer exhibition at higher temperatures, which was in line with Viktorov et al. [5]. Based on the scans, it is assumed that especially longer heating process may result in the appearance of the central cavity in the case of sample 2. This would increase its internal surface and lead to a potentially higher absorption index. Further detailed analysis based on the scans related to the pore size distribution and wall thickness is presented in Figure 3.

The wall thickness analysis results revealed significant variations between all samples (including samples of the same origin—sample 2 and sample 3). In the case of fertilizer-grade ammonium nitrate(V) (Figure 3a), the wall thickness predominantly ranged from 0.05 to 0.40 mm. Notably, walls adjacent to the meandering cracks and channels exhibited finer walls, ranging from 0.1 to 0.2 mm. Sections of the cross-sectioned prill formed by the merging of cracks and channels demonstrated the thinnest walls, measuring between 0.1 to 0.15 mm, exemplified by small “grains” in the central part of the granule or on the upper right segment (indicated in red in Figure 3a). This is likely attributed to the low temperature (heat emission) influence generated by the reagents’ chemical reaction on the ammonium nitrate(V) final product’s surface. Larger sections had a greater surface area, which indicates prolonged decomposition of ammonium nitrate(V) during heating, as observed in the visible “grains” in the right central part of granule 3. Moreover, in contrast to prill ammonium nitrate, fertilizer-grade AN has not been subjected to an additional prilling process that would influence its crystal structure. The pore size distribution for fertilizer-grade ammonium nitrate suggests a range of approximately 0.02 to 0.38 mm, with the maximum pore size (0.38 mm) aligning with the results presented in Figure 3b. The largest pore surfaces measured approximately 0.1 mm^2^, typically within the range of 0.11–0.13 mm. This surface distribution implies that fertilizer-grade ammonium nitrate(V) is characterized by a low area of contact, suggesting a low absorption ratio for Sample 1.

Conversely, porous prill ammonium nitrate (Sample 2) exhibited the finest wall sizes, primarily around 0.05 mm, Figure 3c. This is likely a result of open porosity, coupled with a complex pattern of channels observed in the cross-sectional scans. Similar to fertilizer-grade samples, the finest areas were found near channels and air voids. The pore size distribution, Figure 3d, indicated a range between 0.02 and 0.1 mm, with the peak pore surface (approximately 0.72 mm^2^) observed in the range of 0.02 to 0.04 mm. The Ray method analysis revealed that, considering the deformation and pore surface, Sample 2’s contact surface is larger compared to the fertilizer grade, potentially leading to a greater absorption index.

The smaller prill diameter of ammonium nitrate(V) porous prill, Sample 3, showed a mixture of areas with thinner walls (central part of the prill) and thicker walls (upper green-blue parts of the prill), as depicted in Figure 3e. This distribution likely results from the uneven distribution of internal channels visible under SEM [9,22], potentially due to insufficient thermal treatment time and irregular prill shape. Nevertheless, the peak pore surface indicates a relatively high peak pore surface value of 1.8 mm^2^ (Figure 3f). The pore diameter range was approximately 0.02 to 0.013 mm. Considering the surface distribution, Sample 3 should exhibit a higher contact surface area than Sample 2 (same manufacturer).

Sample 4 ammonium nitrate(V) porous prill exhibited a similar combination of thin and thick walls, akin to ammonium nitrate(V) porous prill Sample 3. This configuration contrasts with the analogous type of ammonium nitrate(V) porous prill, Sample 2, from a different manufacturer. Figure 3g illustrates that around the central cavity, wall thickness reached up to approximately 0.05 mm, gradually increasing with distance from the center to the external part of the prill. It can be inferred that with prolonged temperature treatment, the central cavity would expand. The pore size surface distribution indicates a peak pore surface area exceeding 1.8 mm^2^. This peak value is comparable to Sample 3’s peak surface but significantly higher than Sample 2’s. Furthermore, all porous prill samples demonstrated peak surface pore areas several times greater than that of fertilizer-grade ammonium nitrate(V). Based on the peak surface distribution, Figure 3h, Sample 4 appears to have the largest contact surface, suggesting the highest absorption ratio. The evaluation of absorption capacity was conducted based on the porosimetry analysis, as shown in Table 1.

The results of the surface area (S_BET_) measurements, presented in Table 1, indicate that all ammonium nitrate(V) samples exhibit a low surface area. The highest surface area, approximately 0.466 m^2^·g^−1^, was observed in the porous prill ammonium nitrate(V) sample 4, which was characterized by the presence of a central cavity and extended deformations. In contrast, the lowest surface area of 0.011 m^2^·g^−1^ was found in the fertilizer-grade ammonium nitrate(V). This low surface area can be attributed to the limited number of cracks, and surface deformations, the almost plain surface of the sample, as well as a less developed internal surface, as seen in the tomography scans (Figure 2a). This observation is also consistent with the pore surface distribution shown in Figure 2b.

**Figure 4:**
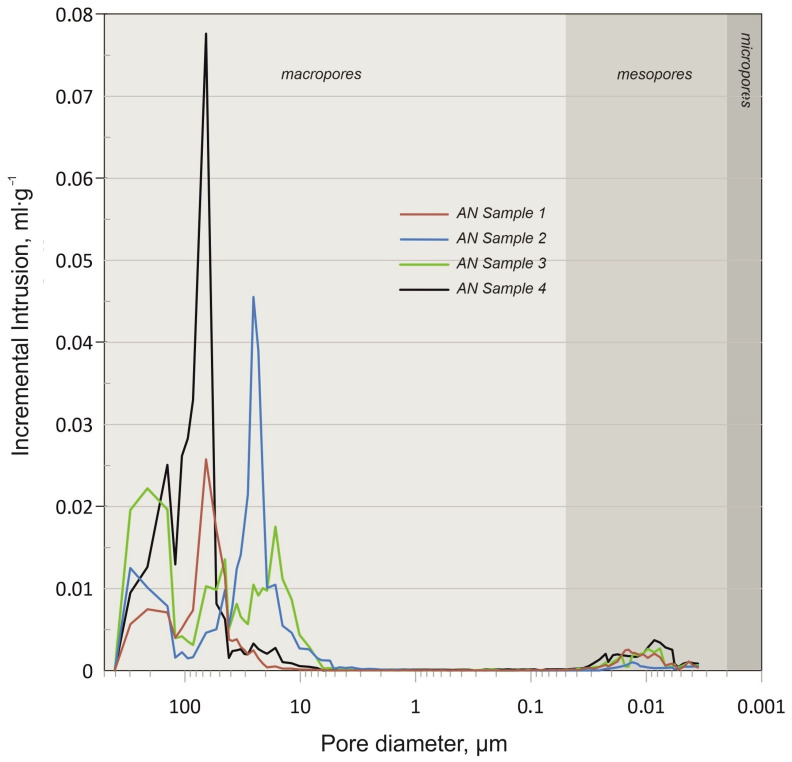
Incremental intrusion vs. pore size. Pore size classification acc. to IUPAC.

For the porous prill samples 2 and 3, a decrease in prill diameter was associated with a slight increase in S_BET_, from 0.098 m^2^·g^−1^ to 0.113 m^2^·g^−1^. This may result from an improved structure, as evidenced by SEM images in [22] and tomography scans in Figure 2b,c, as well as pore surface distributions in Figure 3d,f. The obtained values suggest that, despite the high absorption ratios claimed by manufacturers, the analyzed samples should be considered low-absorption materials. Further evaluation is needed to determine the effectiveness of this method. The results of porosimetry analysis with mercury intrusion are presented in Table 2 and Figure 4.

The results from the mercury intrusion method, as presented in Table 2, indicate a significantly higher S_BET_ surface area compared to the values obtained using the nitrogen adsorption method shown in Table 1. Specifically, the S_BET_ surface area for ammonium nitrate(V) ranges from 4.87 to 18.29 m^2^·g^−1^, with the lowest value of 4.87 m^2^·g^−1^ observed for the fertilizer-grade ammonium nitrate(V). This lower surface area is attributed to the relatively smooth surface and minimal surface deformations, consistent with the SEM observations reported in our previous study [22] and corroborated by other researchers [5,7,9,19].

The mercury intrusion data also reveal that a substantial portion of the incremental intrusion is associated with larger diameter pores (Figure 4), a finding supported by the pore size distribution obtained using the Ray algorithm (Figure 3a). Despite a relatively high porosity of approximately 33.35%, the presence of larger diameter pores leads to a lower active surface area, which in turn results in reduced absorption values.

Conversely, porous prilled ammonium nitrate(V) samples with a grain size of 8 from various manufacturers (Samples 2 and 4) demonstrate a greater S_BET_ surface area than the fertilizer-grade AN. This is likely due to the extended wrinkled structure and the presence of open porosity [5,7,9,18]. The variation in S_BET_ values between AN-PP-1 (11.93 m^2^·g^−1^) and AN-PP-2 (18.28 m^2^·g^−1^) can be attributed to differing manufacturing conditions, which produced distinct porosities of 20.64% and 35.43%, respectively (Table 2). These differences in porosity are further linked to the prevalence of smaller diameter pores, as seen in Figure 4 blue and green lines, which significantly increase the surface area and influence mercury absorption.

Moreover, tomography scans reveal that AN-PP-8-2 exhibits a central cavity, which also contributes to the higher porosity of Sample 4. The smaller diameter prills of AN-PP-7, with a porosity of 31.24%, display a similar SBET value of approximately 12.67 m^2^·g^−1^, comparable to AN-PP-8-1 (11.93 m^2^·g^−1^). The similarity in SBET values can be explained by the physical resemblance between AN-PP-7 and AN-PP-8-1, particularly the absence of a central cavity, as confirmed by SEM analysis [22]. However, the higher number of small diameter pores in AN-PP-7 and AN-PP-8-2 impacts both SBET and porosity, aligning their characteristics more closely with each other.

Sample 3 is primarily characterized by an incremental intrusion dominated by small diameter pores, particularly those below 10 µm. This is consistent with the pore distribution data presented in Figure 3h.

The observed discrepancies in S_BET_ values obtained from mercury intrusion and nitrogen adsorption methods highlight the limitations of nitrogen adsorption for ammonium nitrate(V), as it fails to account for certain surface characteristics. While nitrogen adsorption provides a preliminary indication of SBET trends, accurate surface area values are more reliably obtained through mercury intrusion. Additionally, mercury intrusion offers more precise porosity data compared to tomography, which examines only a single prill or granule per scan, unlike mercury intrusion which assesses a bulk sample. It is important to note that although each sample comprises prills and granules of a specific size distribution, the presence of minor fractions of varying diameters can influence the overall properties. Based on these findings, the mercury intrusion method is recommended for the comprehensive analysis of the porosity properties of ammonium nitrate(V).

The result of the ammonium nitrate(V) absorption capacity based on the fuel oil test is presented in Table 3.

According to Table 3, the lowest oil absorption capacity (3.25%) was observed in the fertilizer-grade ammonium nitrate (AN) sample (Sample 1). This finding aligns with the observation that the limited internal and external crystal structure of AN, which resulted in a low S_BET_ of 4.87 m^2^·g^−1^, significantly influenced the sample’s absorption capacity. In contrast, the highest absorption capacity was noted in the AN-PP-8-2 sample, corresponding to its higher SBET value of 18.29 m^2^·g^−1^. The absorption capacities of AN samples 2 and 3, measured at 9.0% and 11.5%, respectively, were approximately 3–4 times higher than that of the fertilizer-grade AN. This confirms that the crystal surface characteristics of the tested samples [22] play a crucial role in determining absorption capacities. Furthermore, these results are consistent with the findings of Zawadzka-Małota and Maranda, who reported absorption capacities for fertilizer and prilled AN samples ranging from 1.5% to 14.5% [4].

## 4. Conclusions

Research findings indicate that the origin of ammonium nitrate(V) samples significantly affects both wall thickness and specific surface area (S_BET_). Analysis through various porosimetry methods revealed that the highest S_BET_ was observed in the porous prilled ammonium nitrate(V) sample with a grain size of 8, produced by the second manufacturer. This elevated S_BET_ is attributed to the extensive external and internal crystal surface area, as demonstrated in previous studies [21]. In contrast, the lowest SBET was recorded for fertilizer-grade ammonium nitrate(V), which can be explained by its closed porosity and nearly smooth external crystal surface.

However, it is important to note that the S_BET_ values differ significantly between the mercury intrusion method (4.87–18.29 m^2^·g^−1^) and the nitrogen adsorption method (0.011–0.446 m^2^·g^−1^). This discrepancy is likely due to the nitrogen adsorption method’s inability to effectively penetrate the material, highlighting that mercury intrusion is the more reliable method for evaluating the porosity and surface area of ammonium nitrate(V).

Further analysis using the Ray algorithm identified thinner walls in the prilled ammonium nitrate(V) sample with a grain size of 8, manufactured by Yara International ASA (Sample 2). The wall thickness in these prills was consistently below 0.05 mm and uniformly distributed, which could be a result of open porosity and uniform temperature distribution during the prilling process. Conversely, the fertilizer-grade ammonium nitrate(V) (Sample 1) exhibited the thickest walls, likely due to the absence of additional prilling processes. In this case, the fertilizer ammonium nitrate(V) undergoes only the standard heat emission from the synthesis of chemical reagents at the beginning of production.

Both the Ray algorithm and mercury intrusion method confirmed that Sample 4, based on pore size distribution and mercury intrusion volume, had the highest potential absorption ratio, while fertilizer-grade ammonium nitrate(V) exhibited the lowest absorption capacity. These findings are consistent with previous SEM and tomography results [21].

It is worth noting that the porosity results obtained from chemical intrusion methods differ from those presented in our previous studies. This discrepancy can be attributed to the differences between the two methods. Mercury intrusion evaluates multiple prills and granules within a sample, providing a more comprehensive analysis, whereas tomography evaluates only a single prill or granule per scan. Although the majority of each ammonium nitrate (V) sample consists of a defined grain size, the presence of minor fractions with varying cumulative distributions can affect precision. Therefore, in such cases, mercury intrusion provides more objective and reliable results.

The analysis of oil absorption capacities across different ammonium nitrate (AN) samples reveals a clear correlation between specific surface area (SBET) and absorption capacity. The fertilizer-grade AN, with a low SBET of 4.87 m^2^·g^−1^, exhibited the lowest absorption capacity at 3.25%, underscoring the impact of limited internal and external crystal structure. In contrast, the AN-PP-8-2 sample, which had the highest SBET of 18.29 m^2^·g^−1^, demonstrated the greatest absorption capacity. The intermediate absorption capacities observed in samples 2 and 3 further support the significant influence of crystal surface characteristics on absorption performance.

## Figures and Tables

**Table 1:** Results of S_BET_.

Sample	S_BET_, m^2^·g^−1^
1	0.011
2	0.098
3	0.113
4	0.466

**Table 2:** Results of S_BET_ mercury.

Sample	S_BET,_ m^2^·g^−1^	Average Pore Diameter, μm	Porosity, %
1	4.87	0.245	33.35
2	11.93	0.052	20.74
3	12.67	0.084	31.24
4	18.29	0.069	35.43

**Table 3:** Results of absorption capacity.

Sample	Fuel Oil, Absorption Capacity, %
1	3.25
2	9.0
3	11.5
4	13.5

## Data Availability

The original contributions presented in the study are included in the article, further inquiries can be directed to the corresponding author.
